# Toward better evaluation of consumer sleep technologies: a call for rigor, context, and collaboration

**DOI:** 10.1093/sleepadvances/zpaf063

**Published:** 2025-09-17

**Authors:** Massimiliano de Zambotti, Raphael Vallat, Gerald Pho, Cathy Goldstein, Shyamal Patel

**Affiliations:** Ouraring Inc., San Francisco, CA, United States; Oura Health Oy, Oulu, Finland; Ouraring Inc., San Francisco, CA, United States; Sleep Disorders Center, Department of Neurology, University of Michigan, Ann Arbor, MI, United States; Ouraring Inc., San Francisco, CA, United States

Dear Editor,

Consumer sleep technologies (CSTs) have been studied and refined for over a decade, with their adoption steadily growing in research and among patients in sleep disorders centers. These tools can now be used for behavioral monitoring, therapy engagement, and longitudinal sleep tracking. Despite this momentum, barriers to clinical adoption remain—often driven by concerns about data quality, lack of standardized validation, use of proprietary algorithms that lack transparency, regulatory and liability issues, limited integration with electronic health records, challenges in interpreting non-standardized CST outputs, population bias (e.g. suboptimal performance in diverse groups), and entrenched cultural and professional norms.

As CSTs continue to evolve and enter mainstream use, researchers and clinicians have a responsibility to develop evaluation frameworks that are rigorous, technologically current, and contextually grounded. We would like to highlight several examples from recent literature that underscore the urgent need for such shared standardized frameworks to avoid misleading approaches and conclusions related to CST performance and utility.

We observe several recurring issues present across the CST performance evaluation (“validation”) literature. These include inconsistent methodological alignment, outdated device versions, insufficient documentation of device use protocols, and context-blind interpretation of results—all of which merit attention in efforts to establish a shared, rigorous evaluative framework.

The rapid pace of CST development demands transparency around hardware and software versioning, as well as acknowledgment that the time lapse between study launch and publication often means that results apply to earlier device generations and algorithms and may not be directly transferable to newer versions. For example, in a recent 2025 publication by [1], authors evaluated the performance of three ring-based CSTs (Oura Ring Gen3, SleepOn Go2Sleep, Circul Ring) against reference standard polysomnography (PSG) in a diverse clinical population. However, the authors comment on device performance based on data collected in 2022. Since then, newer generations and algorithmic updates have been released—including Oura Ring 4 in 2024 and a sleep staging algorithm update in June 2023. Similar updates apply to Go2Sleep and Circul. Previous investigations have suffered with similar, and in some cases even larger, lags between data collection and the public release of findings. For example, Chinoy et al. [2] evaluated the performance of seven consumer sleep-tracking devices using data collected between 2017 and 2019—a substantial gap, as by the time of publication in 2021, multiple newer generations of the tested models had already been released, limiting the direct applicability of their results to devices in current use. As previously discussed [3], version lag in validation studies is a known challenge and should be contextualized to avoid misleading impressions about a device’s current capabilities. Transparent reporting of device generation, firmware version, and algorithmic update status is essential for accurate interpretation and reproducibility. Encouragingly, on the industry side, initiatives such as firmware-locking and the pre-registration of algorithm releases for research represent important steps toward greater transparency and methodological rigor [4].

Evaluation protocols must match the resolution at which algorithms were trained and perform. CST sleep staging algorithms are typically trained and validated on 30-s epochs, consistent with standard PSG scoring. However, some studies deviate from this time scale, for example, in the study by Herberger et al. [1], the authors compared sleep staging from the Oura Ring 4 to reference-standard PSG using 5-min epochs—likely enabled by access to Oura data via Oura API. This resolution introduces artificial stage classifications and degrades staging accuracy metrics. In other prior CST “validation” studies, device and PSG data have been compared after adjustments for mismatched epoch lengths. For example, Chinoy et al. [2], in their multi-device comparison with PSG, handled devices that output 1-min epochs by rescoring PSG using standard rules applied over 60-s rather than the traditional 30-s epochs. A similar procedure was commonly adopted in earlier “validation” studies (e.g. de Zambotti et al. [5]), when device outputs were accessed at 1-min resolution, following practices inherited from the actigraphy literature. In this approach, two consecutive 30-s PSG epochs were collapsed into a 1-min epoch: if both were scored as “Sleep,” the combined epoch was coded as Sleep; if one or both were scored as “Wake,” the combined epoch was coded as Wake. These issues highlight a broader field-wide challenge: access to appropriately granular data must be prioritized in collaborative performance evaluation research.

CST performance is highly dependent on proper fit and placement, which directly affect signal quality—particularly for photoplethysmography measurements. Several “validation” studies do not follow manufacturer recommendations, often for practical reasons such as comparing multiple devices simultaneously (leading to suboptimal placement) or rotating devices among participants for convenience. Just as importantly, most studies fail to document basic procedures such as fit, placement, or participant instructions. While this constraint is understandable, it surfaces a broader need for the field to establish clear protocols for device fitting and usage. Deviation from manufacturer-recommended wear conditions can meaningfully influence algorithmic performance and should be accounted for in any comparative validation study [3]. Without clear reporting and standardized protocols, it is difficult to know whether poor performance reflects the device itself or study design artifacts.

Strong recommendations against the use of CSTs (e.g. “prohibiting their use in clinical sleep medicine”) should be carefully weighed and contextualized, especially considering how comparable performance tradeoffs are tolerated with other widely used clinical technologies. For instance, positive airway pressure (PAP)-derived apnea–hypopnea index metrics are widely used in clinical decision-making despite inconsistent validation against PSG [6–8]. Likewise, while actigraphy holds regulatory clearance, studies suggest that CSTs outperform it in sleep measurement across several populations. These examples underscore the need for consistency in how we assess and apply performance thresholds across legacy and emerging technologies. There are risks of reinforcing resistance to CST integration in a manner that may be disproportionate to the evidence and misaligned with broader developments in the field. Furthermore, the immediate use case for CST in sleep medicine is to provide collateral information as opposed to acting as a standalone diagnostic tool.

The challenges identified in this letter are systemic. Recent efforts are addressing them. Initiatives such as those led by the Sleep Research Society [9] and the World Sleep Society [10] have highlighted the current capabilities and limitations of consumer sleep-tracking devices, identifying key areas for collaborative advancement. Other efforts have explicitly called for multidisciplinary collaboration [11], engaging stakeholders across academia, clinical practice, and industry. In parallel, guidelines for the development, performance evaluation, and validation of new sleep technologies have been proposed [12]. Efforts to establish industry standards for consumer wearables have been ongoing, driven by organizations such as the Consumer Technology Association, the National Sleep Foundation, and, more recently, the Digital Medicine Society. Simultaneously, several companies—serving both academic research (e.g. health data platforms) and industry (e.g. integration services for wearables and digital health apps)—have attempted to harmonize and standardize wearable-derived outcomes. However, many of these initiatives lack balanced representation from both scientific and industry leaders and have resulted in fragmented efforts with limited visibility and low adoption. A more unified, cross-sector collaboration is urgently needed.

The future of CST evaluation also depends on open science. Access to diverse datasets would help CST companies develop more robust algorithms, while sharing raw biobehavioral data would allow academic researchers to validate device-agnostic, population-specific models—enhancing personalization, equity, and transparency.

We are not at the beginning of the CST journey—we are over a decade in. Resistance to adoption may persist, but so does the opportunity to guide the field toward evidence-based, ethically grounded, and contextually aware integration.

As CSTs become embedded in both consumer and clinical ecosystems, our evaluation strategies must evolve accordingly. Building rigorous, reproducible, and collaborative frameworks is not just beneficial—it is essential.

The following recommendations highlight practical steps the field can take to improve the rigor, context, and collaboration required to evaluate CST:

- **Use established evaluation frameworks**: Apply standardized performance evaluation pipelines (e.g. step-by-step protocols and open-source code now available) to ensure consistency and comparability across studies (see [13], for an example).
- **Explicitly state contextual information**: Always report hardware generation, firmware/software version, and algorithm release (if available). This ensures results are interpretable within their technological and temporal context, which is essential for translational knowledge and reproducibility.
- **Align evaluation with algorithm design**: Test CST outputs using the same resolution and methods (e.g. 30-s epochs for sleep staging) that the algorithms were trained on and intended to produce.
- **Ensure proper use conditions**: Follow manufacturer recommendations for device fitting, placement, and wear protocols whenever possible. If deviations are necessary, clearly document and standardize these procedures to minimize artifacts and reduce risk of misinterpretation.
- **Interpret output with consideration of known limitations**: Benchmark CST performance against established clinical tools and standards (e.g. PSG, actigraphy), while recognizing the intrinsic limitations of these reference measures to set realistic expectations (e.g. imperfect interscorer reliability).
- **Promote transparency and data access**: Encourage open reporting of preprocessing steps and, when possible, access to granular data for independent evaluation.
- **Diversify samples**: Test across heterogeneous populations (age, sex, body mass index, skin tone, comorbidities) to address bias and equity concerns.
- **Avoid overinterpretation**: Do not draw conclusions beyond what the device and data can support; highlight both capabilities and known limitations.

## Notes

*This paper is part of the Consumer Sleep Technology Collection*.
